# Clinical features and neuropsychiatric comorbidities in pediatric patients with tic disorders: a retrospective chart review study from South Korea

**DOI:** 10.1186/s12888-020-03014-z

**Published:** 2021-01-07

**Authors:** Eu Gene Park, Young-Hoon Kim

**Affiliations:** 1 Department of Pediatrics, Incheon St. Mary’s Hospital, College of Medicine, The Catholic University of Korea, 56, Dongsu-ro, Bupyeong-gu, Incheon, 21431 Republic of Korea; 2 Department of Pediatrics, Uijeongbu St. Mary’s Hospital, College of Medicine, The Catholic University of Korea, 271, Cheonbo-ro, Uijeongbu-si, Gyeonggi-do 11765 Republic of Korea

**Keywords:** Tic disorders, Tourette syndrome, Diagnostic and statistical manual of mental disorders, Comorbidity

## Abstract

**Background:**

Tic disorders are childhood-onset neuropsychiatric disorders characterized by multiple motor or vocal tics with frequent comorbidities and a broad spectrum of phenotypic presentations. In this study, we aimed to investigate the clinical characteristics and comorbid neuropsychiatric conditions in pediatric patients with tic disorders.

**Methods:**

We retrospectively reviewed the medical records of 119 pediatric patients (89 males, 30 females) who were diagnosed with tic disorders according to the Diagnostic and Statistical Manual of Mental Disorders, 5th edition (DSM-5) at Uijeongbu St. Mary’s Hospital, Republic of Korea, between January 2012 and July 2019.

**Results:**

The mean age of tic onset was 6.9 years (range, 1–14) and the mean age at diagnosis was 8 years (range, 1–17). The mean lag between tic onset and diagnosis was 13.3 months (range, 0.25–132). The most common, first-presenting tics were eye blinking (50.4%), followed by jaw or lip movement (29.4%) and throat clearing (29.4%). Thirty-seven (31.1%) patients had at least one co-occurring neuropsychiatric disorder at the time of tic diagnosis. Subtypes of tic disorders, types of initial tics, and presence of neuropsychiatric comorbidities were not associated with tic severity. Tic severity was associated with greater functional impairment and tic noticeability (*p* < 0.05). A relatively shorter time to diagnosis was associated with tic severity (Spearman’s ρ = − 0.14, *p* = 0.11).

**Conclusions:**

The evolving nature of tic expression and severity, high prevalence of neuropsychiatric comorbidities, and associated functional impairments emphasize the importance of comprehensive assessment during the disease course for determining and prioritizing goals of treatment.

## Background

Tics are involuntary, stereotyped, nonrhythmic movements or vocalizations that are usually sudden and rapid [1, 2]. A broad spectrum of phenotypes ranging from mild to severe and involving any muscle group characterizes the disorders [3]. Tics are categorized as either motor or vocal, and simple or complex, based on their quality and degree of complexity [4]. They can appear isolated or in combination and may coexist or occur in orchestrated sequences. The average age of onset for tic disorders is 4–6 years, peaking between 10 and 12 years, and declining throughout adolescence [5–7].

The precise etiology of tic disorders remains undetermined; however, there is strong agreement that their presence is associated with both polygenic and environmental factors [8]. Although several susceptibility genes have been suggested, no definitive causative gene mutation or risk allele has been identified, due to various factors such as the phenotypic and genotypic heterogeneity, variations in polygenic burden, rare mutations, epigenetic factors, and gene-environment interactions [9]. Environmental factors such as prenatal exposure, perinatal complications, infections, altered immune regulation, and psychosocial stressors are implicated in the development of tic disorders [10].

Tic disorders are usually associated with mental, behavioral, and developmental comorbidities, with previous studies demonstrating that approximately 80–90% of individuals with tic disorders also present other concurrent neuropsychiatric symptoms [5, 11, 12]. Attention-deficit hyperactivity disorder (ADHD) and obsessive-compulsive disorder (OCD) are the most commonly reported comorbidities, followed by depression [5, 13–15]. O’Hare et al. [16] noted that children with tic disorders and co-occurring neuropsychiatric comorbidities tend to have a lower global quality of life and more severe functional impairment. Considering the impact of associated psychopathology on the well-being of patients, an early diagnosis may allow prompt interventions that can improve quality of life, aid social skills, and facilitate academic achievement [17].

Tic disorders are clinically diagnosed on the basis of a detailed history and a neurological and psychiatric examination [4]. However, diagnostic difficulties can arise in certain circumstances because of the waxing and waning nature of tics and the variability of symptoms [18], which may impede initiating timely and appropriate interventions. The mean estimated interval between onset of tics and diagnosis is relatively long [19], with previous studies reporting a lag period ranging from 3 to 11.9 years [5, 20–22].

In this study, we aimed to investigate the clinical features and comorbid neuropsychiatric disorders in pediatric patients with tic disorders. In addition, we evaluated the effects of demographic and clinical factors on the timing of tic disorder diagnosis and on clinical outcomes.

## Methods

We retrospectively reviewed the medical charts of pediatric patients who were diagnosed with tic disorders according to the Diagnostic and Statistical Manual of Mental Disorders, 5th edition (DSM-5) at Uijeongbu St. Mary’s Hospital, Republic of Korea, between January 2012 and July 2019. Those who did not meet the diagnostic criteria or those who were lost to follow-up were excluded from this study.

Demographic and clinical data included gender, age of tic onset, age at diagnosis, types and duration of tic symptoms, tic severity at the initial clinical visit and at the most recent follow-up, and the presence of neuropsychiatric comorbidities. The diagnosis of neuropsychiatric comorbidities such as ADHD, OCD, depression, and anxiety were documented by a pediatric neurologist or a psychiatrist based on a psychiatric interview, according to the DSM-5 criteria. Developmental delay and intellectual disability were diagnosed based on the Bayley Scales of Infant Development, 3rd edition and Korean Wechsler Intelligence Scale for Children, 4th edition, respectively. The time to diagnosis of tic disorders was defined as the interval between the reported onset of tic symptoms and the timing of tic disorder diagnosis by a physician.

Severity of tics and tic-related impairment were assessed using the translated Korean version of the semi-structured, clinician-rated Yale Global Tic Severity Scale (YGTSS). The Korean version of YGTSS was developed and shown to be a valid and reliable rating scale in a previous study [23]. Tic severity was assessed by summing the total scores of motor and vocal tics on a scale from 0 to 5, in 5 separate dimensions: number, frequency, intensity, complexity, and interference. Total scores were categorized as follows: absence of tics (0), minimal tics (1–9), mild tics (10–19), moderate tics (20–39), and severe tics (40–50). A high tic score was defined as moderate or severe tics (20–50). Tic-related impairment and impact on the individual’s self-esteem, family and peer relationships, and school or job functioning were rated separately on a 6-step ordinal scale (0–60).

All statistical analyses were performed with SPSS 21.0 for Windows (IBM Corp., Armonk, NY, USA). Fisher’s exact test, Pearson’s chi-square test, and Student’s t-test were used to compare the clinical outcomes by baseline demographics. In addition, Spearman’s rank correlation coefficient (ρ) and the Kruskal-Wallis test were used to analyze the relationship between the time to diagnosis of tic disorders and other variables. Univariate and multivariate logistic regression analyses were performed to identify potential predictor variables and to assess the associations between the variables and tic severity. Statistical significance was defined at *p* < 0.05.

Ethical approval for this retrospective study was provided by the Institutional Review Board of Catholic Medical Center (IRB UC19RASI0149).

## Results

### Patient characteristics

We identified 119 pediatric patients (89 males, 30 females) who were diagnosed with tic disorders during the time frame of this study. The mean age of tic onset was 6.9 years (range, 1–14) and the mean age at the time of diagnosis of tic disorders was 8 years (range, 1–17). Of the 119 patients, ninety-two (77.3%) were diagnosed with provisional tic disorder and 19 (16%) with Tourette syndrome. The remaining 8 patients (6.7%) were diagnosed with chronic motor or vocal tic disorder.

The most common, first-presenting tics were eye blinking (50.4%), followed by jaw or lip movement (29.4%) and throat clearing (29.4%). In addition, head-turning or nodding (28.6%), eyeball rolling (26.9%), and arm movement (26.9%) were frequently observed (Table 1). Of the 119 patients, thirty-seven (31.1%) had at least one co-occurring neuropsychiatric disorder at the time of diagnosis of tic disorders. The most common comorbid neuropsychiatric condition was ADHD (10.9%), followed by anxiety disorder (7.6%), developmental delay or intellectual disability (6.7%), and epilepsy (6.7%) (Table 2). Eighty of 119 patients (67.2%) were treated with anti-tic medication: antipsychotics only (*n* = 72), antidepressants only (*n* = 1), and combined antipsychotics and antidepressants (*n* = 7). The remaining 39 patients (32.8%) received behavioral therapy.

**Table 1:** Patient characteristics

	Number of patients (*n* = 119)
Sex ratio (male:female)	89: 30
Age (years) at symptom onset, mean (range)	6.9 (1–14)
Age (years) at diagnosis, mean (range)	8 (1–17)
Time interval (months) between symptom onset and diagnosis, mean (range)	13.3 (0.25–132)
Subtypes of tic disorder	
Provisional tic disorder	92 (77.3%)
Chronic motor or vocal tic disorder	8 (6.7%)
Tourette syndrome	19 (16%)
Initial tic symptoms	
Eye blinking	60 (50.4%)
Jaw/Lip movement	35 (29.4%)
Throat clearing	35 (29.4%)
Head turning/Nodding	34 (28.6%)
Eyeball rolling	32 (26.9%)
Arm movement	32 (26.9%)
Palilalia	25 (21%)
Shoulder movement	23 (19.3%)
Grimacing	21 (17.6%)
Leg movement	19 (16%)
Sniffling	18 (15.1%)
Coughing	15 (12.6%)
Trunk movement	14 (11.8%)
Sighing	5 (4.2%)
Echolalia	4 (3.4%)
Coprolalia	3 (2.5%)
Grunting	3 (2.5%)
Whistling	2 (1.7%)
Spitting	1 (0.8%)

**Table 2:** Comorbid neuropsychiatric disorders

	Number of patients (*n* = 119)
Attention-deficit hyperactivity disorder	13 (10.9%)
Anxiety disorder	9 (7.6%)
Developmental delay/Intellectual disability	8 (6.7%)
Epilepsy	8 (6.7%)
Self-injurious behavior	4 (3.4%)
Enuresis	3 (2.5%)
Obsessive-compulsive disorder	1 (0.8%)
Depression	1 (0.8%)
Adjustment disorder	1 (0.8%)
Night terror	1 (0.8%)

### Tic severity and associated impairment

Seventy-six (63.9%) children in the sample were reported to have mild symptoms, thirty (25.2%) had moderate symptoms, and the remaining thirteen (10.9%) had severe symptoms. A comparison of demographic and clinical characteristics of patients with mild tics and those with moderate to severe tics is shown in Table 3. No significant differences were found between patients diagnosed with mild tics compared to moderate/severe tics with regard to gender, age at symptom onset, age at diagnosis, or time between symptom onset and diagnosis. Subtypes of tic disorders, types of initial tics, and presence of neuropsychiatric comorbidities were not associated with tic severity. Based on multivariate regression analysis, gender, age at symptom onset, age at diagnosis, time between symptom onset and diagnosis, subtypes of tic disorders, types of initial tics, and presence of neuropsychiatric comorbidities, were not significantly related to tic severity (Table 4). However, patients with moderate to severe tics were significantly more likely to have tics that were noticeable to strangers and that interfered with their daily functioning, compared to those with mild tics. When the patients were divided into three groups according to subtypes of tic disorders, no significant differences in gender, age at symptom onset, age at diagnosis, time between symptom onset and diagnosis, types of initial tics, and presence of neuropsychiatric comorbidities were observed between patients with different tic severities. Compared to those with mild tics, patients with provisional tic disorder and those with moderate to severe tics, were significantly more likely to have tics that were noticeable to strangers and that interfered with their daily functioning.

**Table 3:** Comparison of demographic and clinical characteristics of patients based on tic severity

	Patients with mild tics (*n* = 76)	Patients with moderate to severe tics (*n* = 43)	*P* value
Gender (Male)^a^	55 (72.4%)	34 (79.1%)	0.41
Age (years) at symptom onset, mean (range)^b^	6.9 (1.2–14.5)	7.9 (1.3–14.3)	0.07
Age (years) at diagnosis, mean (range)^b^	7.9 (1.7–17.2)	9.1 (2.1–17.4)	0.08
Time interval (months) between symptom onset and diagnosis, mean (range)^b^	12.9 (0.25–96)	13.9 (0.25–132)	0.81
Subtypes of tic disorder^a^			
Provisional tic disorder	59 (77.6%)	33 (76.7%)	0.91
Chronic motor or vocal tic disorder	6 (7.9%)	2 (4.7%)	0.49
Tourette syndrome	11 (14.5%)	8 (18.6%)	0.55
Initial tic symptoms			
Motor tics^a^	38 (50%)	14 (32.6%)	0.06
Vocal tics^c^	4 (5.3%)	4 (9.3%)	0.45
Motor + vocal tics^a^	34 (44.7%)	25 (58.1%)	0.16
Presence of neuropsychiatric comorbidities^a^	21 (27.6%)	16 (37.2%)	0.27
Tics are noticeable to strangers^a^	38 (50%)	31 (72.1%)	0.01
Tics interfere with functioning^a^	24 (31.6%)	30 (69.8%)	< 0.05

^a^Chi-square test was used

^b^Independent t-test was used

^c^Fisher’s exact test was used

**Table 4:** Multivariate analysis of variables associated with tic severity

	Odds ratio (95% CI)	*P* value
Gender	0.67 (0.24–1.88)	0.45
Age at symptom onset	0.09 (0–1,059,041.33)	0.77
Age at diagnosis	11.79 (0–131,775,769.7)	0.76
Time interval between symptom onset and diagnosis	0.81 (0.21–3.16)	0.77
Subtypes of tic disorder		
Provisional tic disorder	1	0.37
Chronic motor or vocal tic disorder	1.43 (0.39–5.21)	0.58
Tourette syndrome	0.38 (0.04–3.37)	0.39
Initial tic symptoms		
Motor tics	1	0.77
Vocal tics	1.47 (0.26–8.13)	0.65
Motor + vocal tics	1.42 (0.50–3.97)	0.50
Presence of neuropsychiatric comorbidities	1.43 (0.58–3.52)	0.43

### Factors influencing time to diagnosis

The mean lag time from when tics were first noticed to diagnosis of tic disorders was 13.3 months (range, 0.25–132). Gender differences in lag time to diagnosis were observed, 10.0 months for females compared to 8.0 months for males (*p* = 0.05). No significant difference was found in the time to diagnosis between patients with the presence of neuropsychiatric comorbidities, and those without (*p* = 0.91). In addition, age at symptom onset was not correlated with the time to diagnosis (Spearman’s ρ = − 0.14, *p* = 0.11). The mean time to diagnosis was shorter in patients whose initial tics were motor tics only, compared to those with vocal tics only and combined motor and vocal tics, with a mean of 5.6 months (range, 0.25–24), 21.3 months (range, 0.25–96), and 18.9 months (range, 0.25–132), respectively (*p* < 0.05). Tic severity was negatively correlated with diagnostic lag time (Spearman’s ρ = − 0.18, *p* < 0.05), that is, the more severe the tics, the less time that elapsed before a diagnosis.

### Predictors of follow-up outcomes

The patients were followed up for an average of 20.2 months (range, 0.1–88.5) after the diagnosis of tic disorders. Of 119 patients, ninety-nine (83.2%) reported a greater than 50% reduction in tic severity, and twenty (16.8%) reported no changes in the tic symptoms at follow-up. To determine the factors related to favorable outcomes, we compared the characteristics of patients who achieved more than 50% reduction in symptoms with those who had no change in tic severity. Gender, age of onset, age at diagnosis, time interval between onset and diagnosis, subtypes of tic disorders, initial symptoms, tic severity at diagnosis, presence of neuropsychiatric comorbidities, and treatment utilization did not influence likelihood of favorable outcomes (Table 5). Based on multivariate regression analysis, gender, age of onset, age at diagnosis, time interval between onset and diagnosis, subtypes of tic disorders, initial symptoms, tic severity at diagnosis, presence of neuropsychiatric comorbidities, and treatment utilization, were not significantly related to clinical outcomes (Table 6). When the patients were divided into three groups according to subtypes of tic disorders, no significant differences regarding gender, age of onset, age at diagnosis, time interval between onset and diagnosis, initial tic symptoms, tic severity at diagnosis, presence of neuropsychiatric comorbidities, or treatment utilization were observed between patients who showed different outcomes.

**Table 5:** Comparison of demographic and clinical features based on clinical outcomes

	Patients with > 50% reduction in tics (*n* = 99)	Patients with no changes in tics (*n* = 20)	*P* value
Gender (Male)^a^	74 (74.7%)	15 (75%)	0.98
Age (years) at symptom onset, mean (range)^b^	7.1 (1.2–13.9)	8.2 (1.7–14.5)	0.12
Age (years) at diagnosis, mean (range)^b^	8.2 (1.7–17.4)	9.1 (2.4–14.9)	0.26
Time interval (months) between symptom onset and diagnosis, mean (range)^b^	13.7 (0.25–132)	11.2 (0.25–60)	0.63
Subtypes of tic disorder^a^			
Provisional tic disorder	77 (77.8%)	15 (75%)	0.78
Chronic motor or vocal tic disorder	7 (7.1%)	1 (5%)	0.73
Tourette syndrome	15 (15.1%)	4 (20%)	0.58
Initial tic symptoms^a^			
Motor tics	44 (44.4%)	8 (40%)	0.71
Vocal tics	8 (8.1%)	0	0.18
Motor + vocal tics	47 (47.5%)	12 (60%)	0.30
YGTSS at diagnosis, mean (range)^b^	12.6 (0–50)	15 (0–30)	0.37
Presence of neuropsychiatric comorbidities^a^	32 (32.3%)	5 (25%)	0.51
Treatment utilization^a^			
Medication	66 (66.7%)	14 (70%)	0.77
Nonmedical treatment	33 (33.3%)	6 (30%)	0.77

*YGTSS* Yale Global Tic Severity Scale

^a^Chi-square test was used

^b^Independent t-test was used

**Table 6:** Multivariate analysis of variables associated with clinical outcomes

	Odds ratio (95% CI)	*P* value
Gender	1.25 (0.38–4.13)	0.70
Age at symptom onset	0.08 (0–7,705,330.35)	0.79
Age at diagnosis	13.17 (0–1,169,765,988)	0.78
Time interval between symptom onset and diagnosis	0.80 (0.17–3.67)	0.77
Subtypes of tic disorder		
Provisional tic disorder	1	0.99
Chronic motor or vocal tic disorder	0.98 (0.22–4.25)	0.98
Tourette syndrome	0.95 (0.06–13.25)	0.97
Initial tic symptoms		
Motor tics	1	0.74
Vocal tics	0	0.99
Motor + vocal tics	1.58 (0.48–5.10)	0.44
YGTSS at diagnosis	1.02 (0.97–1.07)	0.39
Presence of neuropsychiatric comorbidities	0.64 (0.19–2.07)	0.46
Treatment utilization	1.12 (0.34–3.66)	0.84

*YGTSS* Yale Global Tic Severity Scale

## Discussion

Isolated and transient tics are fairly common in childhood, ranging from 11 to 20% in different studies [24–26]. Diagnostic issues may arise in certain circumstances due to the waxing and waning nature and heterogeneity of tic symptom presentation [18]. Additionally, only a few studies have evaluated how many children with transient tics would become cases of Tourette syndrome or chronic tic disorder over time [27]. Therefore, in this study we tried to delineate the clinical features of tic disorders and characterize the demographic and clinical factors influencing the timing of tic disorder diagnosis and clinical outcomes.

We found that the mean age of 6.9 years for the onset of tics was higher than that reported in other studies, which ranged between 4 and 6 years for onset [5–7]. Mild tic symptoms may initially go unrecognized by caregivers and become noticeable only with increasing severity, which may account for the higher mean age at onset in this study. Consistent with previous studies by Robertson et al. [28], we found a higher proportion of tic disorders in males than females in our study. With regard to initial tic symptoms, eye blinking was observed most frequently, accounting for more than half of tic symptoms in patients (50.4%). Previous studies have also noted that tics usually begin in the head and face, with eye blinking being the first and the most common tic [29, 30].

Children with tic disorders have a higher likelihood of concomitant neuropsychiatric conditions [31]. An analysis of shared heritability in 23 different neurologic and psychiatric disorders demonstrated that a significant proportion of polygenic heritability in tic disorders is shared with OCD and ADHD [32]. Our results identified ADHD as the most commonly reported comorbid neuropsychiatric disorder, a finding consistent with previous research [33]. Tics are often not the most enduring or impairing problem in children with tic disorders; other coexisting neuropsychiatric conditions are often a greater source of impairment than the tics themselves [34]. Comorbid ADHD symptoms may have an undesirable impact on social, academic, and behavioral functions and can negatively impact quality of life and global psychosocial functioning [35–37]. Carter et al. [38] found that children with ADHD and tic disorders have more behavioral problems and poorer social adaptation compared to children with tic disorders only. Moreover, it has been postulated that the presence of ADHD in patients with tic disorders is correlated with higher rates of other neuropsychiatric comorbidities, such as OCD, anxiety disorders, anger control disorders, mood disorders, and personality disorders [5]. Our data noted a similar trend in that children with moderate to severe tics had more co-occurring neuropsychiatric disorders than those with mild tics, although the differences were not statistically significant. At a more general level, our results align with previous research showing that children with tic disorders may have increased healthcare needs and require supplemental mental health or educational services and support [39]. Therefore, clinical assessment of pediatric patients with tic disorders warrants a thorough evaluation for coexisting ADHD and other neuropsychiatric problems.

In our study, a relatively short lag time to diagnosis following the onset of tic symptoms was observed, with a mean duration of 13.3 months. This lag is significantly shorter than the 3 to 8 years delay reported in previous studies [5, 21]. The shorter time to diagnosis identified in our study is comparable to findings from more recent studies [19, 40]. In addition, time lag from when tics were first noticed to diagnosis was negatively correlated with tic severity. Growing awareness of tic symptoms among physicians and the availability of health information to patients and their families may be important factors in shortening the time to diagnosis [19]. Timely diagnosis of tic disorders may enable patients and their families to have access to optimized medical, psychological, and educational treatment and other support services [40].

We also found that patients with motor tics as the initial manifestation experienced less time to diagnosis than those with vocal tics. Patients with only vocal tics experienced relatively longer times to diagnosis compared to those with only motor tics or combined motor and vocal tics. Because throat clearing, sniffling, coughing, and grunting are the most common types of vocal tics, patients with vocal tics can often be misdiagnosed as having common pediatric conditions such as a cold, asthma, or allergy, which may contribute to diagnostic delay [4, 19].

With regard to follow-up outcomes, the vast majority of patients (83.2%) reported improvement in their tics over time in our study. Tics reach their apex early in the second decade of life and then usually improve during adolescence [30]. According to Bloch et al. [6], more than three-quarters of children with childhood tic symptoms had fewer tics or were completely tic-free at follow-up, while less than a quarter had moderate or greater tics at follow-up. Though questions regarding the clinical predictive factors of follow-up outcomes arise frequently in clinical practice, previous studies have not successfully identified reliable predictors of follow-up outcomes. Prognostic issues can be difficult to address clinically because tic disorders have a complex range of possible symptom combinations and comorbidities [41]. In our study, we attempted to delineate the demographic and clinical factors that could be used to predict the future course of tic disorders. There were, however, no associations between future tic severity and gender, age of tic onset or diagnosis, lag time to diagnosis, subtypes of tic disorders, types of initial tic symptoms, tic severity at diagnosis, presence of neuropsychiatric comorbidities, or treatment utilization. Although we did not uncover robust clinical predictors of the course of tic disorders, our findings may provide a useful platform for further long-term follow-up studies regarding this issue.

Our study is limited by its retrospective nature. Drawing our patient sample from a single referral center likely influenced the lack of statistical significance. Thus, larger prospective studies or collaborative trials are warranted to elucidate the complex interaction of tic severity, comorbidities, and tic-related impairment and reveal the predictors of long-term follow-up outcomes.

## Conclusions

The findings of this study suggest that motor tics as the initial manifestation and greater tic severity are associated with a shorter time to diagnosis. Although robust clinical predictors of the course of tic disorders were not identified, our findings extend previous research and provide potential explanations regarding the clinical features or neuropsychiatric comorbidities that influence the time to diagnosis and predict clinical outcomes in pediatric patients with tic disorders. We also noted heterogeneity in the initial presentation of tics and a higher likelihood of concomitant neuropsychiatric conditions. Thus, this study provides a better understanding and greater insight into the complexities of tic disorders. The evolving nature of tic expression and severity, high prevalence of neuropsychiatric comorbidities, and associated functional impairments reinforce the importance of comprehensive assessment during the disease course for determining and prioritizing goals of treatment. Furthermore, better recognition among parents, teachers, and health care providers of potential challenges related to either tic disorders or co-occurring conditions would help to best support the patient’s future health and well-being.

## Data Availability

The datasets used and analyzed during the current study are available from the corresponding author on reasonable request.

## Abbreviations

DSM-5: Diagnostic and Statistical Manual of Mental Disorders, 5th edition
ADHD: Attention-deficit hyperactivity disorder
OCD: Obsessive-compulsive disorder
YGTSS: Yale Global Tic Severity Scale

## References

[1] Jankovic J (1997). Tourette syndrome. Phenomenology and classification of tics. Neurol Clin.

[2] Wolicki SB, Bitsko RH, Danielson ML, Holbrook JR, Zablotsky B, Walkup JT, Woods DW, Mink JW (2019). Children with Tourette syndrome in the United States: parent-reported diagnosis, co-occurring disorders, severity, and influence of activities on tics. J Dev Behav Pediatr.

[3] Groth C, Debes NM, Skov L (2017). Phenotype development in adolescents with Tourette syndrome: a large clinical longitudinal study. J Child Neurol.

[4] Ludolph AG, Roessner V, Münchau A, Müller-Vahl K (2012). Tourette syndrome and other tic disorders in childhood, adolescence and adulthood. Dtsch Arztebl Int.

[5] Freeman RD, Fast DK, Burd L, Kerbeshian J, Robertson MM, Sandor P (2000). An international perspective on Tourette syndrome: selected findings from 3,500 individuals in 22 countries. Dev Med Child Neurol.

[6] Bloch MH, Leckman JF (2009). Clinical course of Tourette syndrome. J Psychosom Res.

[7] Burd L, Kerbeshian PJ, Barth A, Klug MG, Avery PK, Benz B (2001). Long-term follow-up of an epidemiologically defined cohort of patients with Tourette syndrome. J Child Neurol.

[8] Singer HS (2019). Tics and Tourette syndrome. Continuum (Minneap Minn).

[9] Georgitsi M, Willsey AJ, Mathews CA, State M, Scharf JM, Paschou P (2016). The genetic etiology of Tourette syndrome: large-scale collaborative efforts on the precipice of discovery. Front Neurosci.

[10] Hoekstra PJ, Dietrich A, Edwards MJ, Elamin I, Martino D (2013). Envinronmental factors in Tourette syndrome. Neurosci Biobehav Rev.

[11] Bitsko RH, Holbrook JR, Visser SN, Mink JW, Zinner SH, Ghandour RM, Blumberg SJ (2014). A national profile of Tourette syndrome, 2011-2012. J Dev Behav Pediatr.

[12] Hirschtritt ME, Lee PC, Pauls DL, Dion Y, Grados MA, Illmann C, King RA, Sandor P, McMahon WM, Lyon GJ, Cath DC, Kurlan R, Robertson MM, Osiecki L, Scharf JM, Mathews CA, Tourette Syndrome Association International Consortium for Genetics (2015). Lifetime prevalence, age of risk, and genetic relationships of comorbid psychiatric disorders in Tourette syndrome. JAMA Psychiatry.

[13] Ganos C, Münchau A, Bhatia KP (2014). The semiology of tics, Tourette’s, and their associations. Mov Disord Clin Pract.

[14] Robertson MM (2000). Tourette syndrome, associated conditions and the complexities of treatment. Brain.

[15] Robertson MM (2006). Mood disorders and Gilles de la Tourette’s syndrome: an update on prevalence, etiology, comorbidity, clinical associations, and implications. J Psychosom Res.

[16] O'Hare D, Helmes E, Reece J, Eapen V, McBain K (2016). The differential impact of Tourette’s syndrome and comorbid diagnosis on the quality of life and functioning of diagnosed children and adolescents. J Child Adolesc Psychiatr Nurs.

[17] Peterson BS, Cohen DJ (1998). The treatment of Tourette’s syndrome: multimodal, developmental intervention. J Clin Psychiatry.

[18] Bruun RD, Budman CL (1997). The course and prognosis of Tourette syndrome. Neurol Clin.

[19] Shilon Y, Pollak Y, Benarroch F, Gross-Tsur V (2008). Factors influencing diagnosis delay in children with Tourette syndrome. Eur J Paediatr Neurol.

[20] Shapiro AK, Shapiro E, Wayne HL (1973). The symptomatology and diagnosis of Gilles de la Tourette’s syndrome. J Am Acad Child Psychiatry.

[21] Wand R, Shady G, Broder R, Furer P, Staley D (1992). Tourette syndrome: issues in diagnosis. Neurosci Biobehav Rev.

[22] Khalifa N, von Knorring AL (2005). Tourette syndrome and other tic disorders in a total population of children: clinical assessment and background. Acta Paediatr.

[23] Chung SJ, Lee JS, Yoo TI, Koo YJ, Jeon SI, Kim BS, Hong KE (1998). Development of the Korean version of Yale Global Tic Severity Scale: a validity and reliability study. J Korean Neuropsychiatr Assoc.

[24] Cubo E, Gabriel y Galán JM, Villaverde VA, Velasco SS, Benito VD, Macarrón JV, Guevara JC, Louis ED, Benito-León J (2011). Prevalence of tics in schoolchildren in central Spain: a population-based study. Pediatr Neurol.

[25] Kurlan R, McDermott MP, Deeley C, Como PG, Brower C, Eapen S, Andresen EM, Miller B (2001). Prevalence of tics in schoolchildren and association with placement in special education. Neurology.

[26] Linazasoro G, Van Blercom N, de Zárate CO (2006). Prevalence of tic disorder in two schools in the Basque country: results and methodological caveats. Mov Disord.

[27] Scahill L, Specht M, Page C (2014). The prevalence of tic disorders and clinical characteristics in children. J Obsessive Compuls Relat Disord.

[28] Robertson MM (1994). Annotation: Gilles de la Tourette syndrome--an update. J Child Psychol Psychiatry.

[29] Bruun RD (1984). Gilles de la Tourette’s syndrome. An overview of clinical experience. J Am Acad Child Psychiatry.

[30] Leckman JF, Zhang H, Vitale A, Lahnin F, Lynch K, Bondi C, Kim YS, Peterson BS. Course of tic severity in Tourette syndrome: the first two decades. Pediatrics. 1998;102:14–9. 10.1542/peds.102.1.14 9651407

[31] Leckman JF. Tourette’s syndrome. Lancet. 2002;360:1577–86. 10.1016/S0140-6736(02)11526-1 12443611

[32] Brainstorm Consortium, Anttila V, Bulik-Sullivan B, Finucane HK, Walters RK, Bras J, Duncan L, Escott-Price V, Falcone GJ, Gormley P, Malik R, Patsopoulos NA, Ripke S, Wei Z, Yu D, Lee PH, Turley P, Grenier-Boley B, Chouraki V, Kamatani Y, Berr C, Letenneur L, Hannequin D, Amouyel P, Boland A, Deleuze JF, Duron E, Vardarajan BN, Reitz C, Goate AM, Huentelman MJ, Kamboh MI, Larson EB, Rogaeva E, St George-Hyslop P, Hakonarson H, Kukull WA, Farrer LA, Barnes LL, Beach TG, Demirci FY, Head E, Hulette CM, Jicha GA, Kauwe JSK, Kaye JA, Leverenz JB, Levey AI, Lieberman AP, Pankratz VS, Poon WW, Quinn JF, Saykin AJ, Schneider LS, Smith AG, Sonnen JA, Stern RA, Van Deerlin VM, Van Eldik LJ, Harold D, Russo G, Rubinsztein DC, Bayer A, Tsolaki M, Proitsi P, Fox NC, Hampel H, Owen MJ, Mead S, Passmore P, Morgan K, Nöthen MM, Rossor M, Lupton MK, Hoffmann P, Kornhuber J, Lawlor B, McQuillin A, Al-Chalabi A, Bis JC, Ruiz A, Boada M, Seshadri S, Beiser A, Rice K, van der Lee SJ, De Jager PL, Geschwind DH, Riemenschneider M, Riedel-Heller S, Rotter JI, Ransmayr G, Hyman BT, Cruchaga C, Alegret M, Winsvold B, Palta P, Farh KH, Cuenca-Leon E, Furlotte N, Kurth T, Ligthart L, Terwindt GM, Freilinger T, Ran C, Gordon SD, Borck G, Adams HHH, Lehtimäki T, Wedenoja J, Buring JE, Schürks M, Hrafnsdottir M, Hottenga JJ, Penninx B, Artto V, Kaunisto M, Vepsäläinen S, Martin NG, Montgomery GW, Kurki MI, Hämäläinen E, Huang H, Huang J, Sandor C, Webber C, Muller-Myhsok B, Schreiber S, Salomaa V, Loehrer E, Göbel H, Macaya A, Pozo-Rosich P, Hansen T, Werge T, Kaprio J, Metspalu A, Kubisch C, Ferrari MD, Belin AC, van den Maagdenberg AMJM, Zwart JA, Boomsma D, Eriksson N, Olesen J, Chasman DI, Nyholt DR, Avbersek A, Baum L, Berkovic S, Bradfield J, Buono RJ, Catarino CB, Cossette P, De Jonghe P, Depondt C, Dlugos D, Ferraro TN, French J, Hjalgrim H, Jamnadas-Khoda J, Kälviäinen R, Kunz WS, Lerche H, Leu C, Lindhout D, Lo W, Lowenstein D, McCormack M, Møller RS, Molloy A, Ng PW, Oliver K, Privitera M, Radtke R, Ruppert AK, Sander T, Schachter S, Schankin C, Scheffer I, Schoch S, Sisodiya SM, Smith P, Sperling M, Striano P, Surges R, Thomas GN, Visscher F, Whelan CD, Zara F, Heinzen EL, Marson A, Becker F, Stroink H, Zimprich F, Gasser T, Gibbs R, Heutink P, Martinez M, Morris HR, Sharma M, Ryten M, Mok KY, Pulit S, Bevan S, Holliday E, Attia J, Battey T, Boncoraglio G, Thijs V, Chen WM, Mitchell B, Rothwell P, Sharma P, Sudlow C, Vicente A, Markus H, Kourkoulis C, Pera J, Raffeld M, Silliman S, Boraska Perica V, Thornton LM, Huckins LM, William Rayner N, Lewis CM, Gratacos M, Rybakowski F, Keski-Rahkonen A, Raevuori A, Hudson JI, Reichborn-Kjennerud T, Monteleone P, Karwautz A, Mannik K, Baker JH, O'Toole JK, Trace SE, Davis OSP, Helder SG, Ehrlich S, Herpertz-Dahlmann B, Danner UN, van Elburg AA, Clementi M, Forzan M, Docampo E, Lissowska J, Hauser J, Tortorella A, Maj M, Gonidakis F, Tziouvas K, Papezova H, Yilmaz Z, Wagner G, Cohen-Woods S, Herms S, Julià A, Rabionet R, Dick DM, Ripatti S, Andreassen OA, Espeseth T, Lundervold AJ, Steen VM, Pinto D, Scherer SW, Aschauer H, Schosser A, Alfredsson L, Padyukov L, Halmi KA, Mitchell J, Strober M, Bergen AW, Kaye W, Szatkiewicz JP, Cormand B, Ramos-Quiroga JA, Sánchez-Mora C, Ribasés M, Casas M, Hervas A, Arranz MJ, Haavik J, Zayats T, Johansson S, Williams N, Dempfle A, Rothenberger A, Kuntsi J, Oades RD, Banaschewski T, Franke B, Buitelaar JK, Arias Vasquez A, Doyle AE, Reif A, Lesch KP, Freitag C, Rivero O, Palmason H, Romanos M, Langley K, Rietschel M, Witt SH, Dalsgaard S, Børglum AD, Waldman I, Wilmot B, Molly N, Bau CHD, Crosbie J, Schachar R, Loo SK, McGough JJ, Grevet EH, Medland SE, Robinson E, Weiss LA, Bacchelli E, Bailey A, Bal V, Battaglia A, Betancur C, Bolton P, Cantor R, Celestino-Soper P, Dawson G, De Rubeis S, Duque F, Green A, Klauck SM, Leboyer M, Levitt P, Maestrini E, Mane S, De-Luca DM, Parr J, Regan R, Reichenberg A, Sandin S, Vorstman J, Wassink T, Wijsman E, Cook E, Santangelo S, Delorme R, Rogé B, Magalhaes T, Arking D, Schulze TG, Thompson RC, Strohmaier J, Matthews K, Melle I, Morris D, Blackwood D, McIntosh A, Bergen SE, Schalling M, Jamain S, Maaser A, Fischer SB, Reinbold CS, Fullerton JM, Guzman-Parra J, Mayoral F, Schofield PR, Cichon S, Mühleisen TW, Degenhardt F, Schumacher J, Bauer M, Mitchell PB, Gershon ES, Rice J, Potash JB, Zandi PP, Craddock N, Ferrier IN, Alda M, Rouleau GA, Turecki G, Ophoff R, Pato C, Anjorin A, Stahl E, Leber M, Czerski PM, Cruceanu C, Jones IR, Posthuma D, Andlauer TFM, Forstner AJ, Streit F, Baune BT, Air T, Sinnamon G, Wray NR, MacIntyre DJ, Porteous D, Homuth G, Rivera M, Grove J, Middeldorp CM, Hickie I, Pergadia M, Mehta D, Smit JH, Jansen R, de Geus E, Dunn E, Li QS, Nauck M, Schoevers RA, Beekman AT, Knowles JA, Viktorin A, Arnold P, Barr CL, Bedoya-Berrio G, Bienvenu OJ, Brentani H, Burton C, Camarena B, Cappi C, Cath D, Cavallini M, Cusi D, Darrow S, Denys D, Derks EM, Dietrich A, Fernandez T, Figee M, Freimer N, Gerber G, Grados M, Greenberg E, Hanna GL, Hartmann A, Hirschtritt ME, Hoekstra PJ, Huang A, Huyser C, Illmann C, Jenike M, Kuperman S, Leventhal B, Lochner C, Lyon GJ, Macciardi F, Madruga-Garrido M, Malaty IA, Maras A, McGrath L, Miguel EC, Mir P, Nestadt G, Nicolini H, Okun MS, Pakstis A, Paschou P, Piacentini J, Pittenger C, Plessen K, Ramensky V, Ramos EM, Reus V, Richter MA, Riddle MA, Robertson MM, Roessner V, Rosário M, Samuels JF, Sandor P, Stein DJ, Tsetsos F, Van Nieuwerburgh F, Weatherall S, Wendland JR, Wolanczyk T, Worbe Y, Zai G, Goes FS, McLaughlin N, Nestadt PS, Grabe HJ, Depienne C, Konkashbaev A, Lanzagorta N, Valencia-Duarte A, Bramon E, Buccola N, Cahn W, Cairns M, Chong SA, Cohen D, Crespo-Facorro B, Crowley J, Davidson M, DeLisi L, Dinan T, Donohoe G, Drapeau E, Duan J, Haan L, Hougaard D, Karachanak-Yankova S, Khrunin A, Klovins J, Kučinskas V, Lee Chee Keong J, Limborska S, Loughland C, Lönnqvist J, Maher B, Mattheisen M, McDonald C, Murphy KC, Nenadic I, van Os J, Pantelis C, Pato M, Petryshen T, Quested D, Roussos P, Sanders AR, Schall U, Schwab SG, Sim K, So HC, Stögmann E, Subramaniam M, Toncheva D, Waddington J, Walters J, Weiser M, Cheng W, Cloninger R, Curtis D, Gejman PV, Henskens F, Mattingsdal M, Oh SY, Scott R, Webb B, Breen G, Churchhouse C, Bulik CM, Daly M, Dichgans M, Faraone SV, Guerreiro R, Holmans P, Kendler KS, Koeleman B, Mathews CA, Price A, Scharf J, Sklar P, Williams J, Wood NW, Cotsapas C, Palotie A, Smoller JW, Sullivan P, Rosand J, Corvin A, Neale BM, Schott JM, Anney R, Elia J, Grigoroiu-Serbanescu M, Edenberg HJ, Murray R. Analysis of shared heritability in common disorders of the brain. Science. 2018;360(6395):eaap8757. 10.1126/science.aap8757 PMC6097237 29930110

[33] Khalifa N, von Knorring AL (2006). Psychopathology in a Swedish population of school children with tic disorders. J Am Acad Child Adolesc Psychiatry.

[34] Leckman JF, King RA, Bloch MH (2014). Clinical features of Tourette syndrome and tic disorders. J Obsessive Compuls Relat Disord.

[35] Cavanna AE, Servo S, Monaco F, Robertson MM (2009). The behavioral spectrum of Gilles de la Tourette syndrome. J Neuropsychiatry Clin Neurosci.

[36] Robertson MM (2006). Attention deficit hyperactivity disorder, tics and Tourette's syndrome: the relationship and treatment implications. A commentary. Eur Child Adolesc Psychiatry.

[37] Sukhodolsky DG, Scahill L, Zhang H, Peterson BS, King RA, Lombroso PJ, Katsovich L, Findley D, Leckman JF (2003). Disruptive behavior in children with Tourette’s syndrome: association with ADHD comorbidity, tic severity, and functional impairment. J Am Acad Child Adolesc Psychiatry.

[38] Carter AS, O'Donnell DA, Schultz RT, Scahill L, Leckman JF, Pauls DL (2000). Social and emotional adjustment in children affected with Gilles de la Tourette’s syndrome: associations with ADHD and family functioning. Attention Deficit Hyperactivity Disorder. J Child Psychol Psychiatry.

[39] Claussen AH, Bitsko RH, Holbrook JR, Bloomfield J, Giordano K (2018). Impact of Tourette syndrome on school measures in a nationally representative sample. J Dev Behav Pediatr.

[40] Hollis C, Pennant M, Cuenca J, Glazebrook C, Kendall T, Whittington C, Stockton S, Larsson L, Bunton P, Dobson S, Groom M, Hedderly T, Heyman I, Jackson GM, Jackson S, Murphy T, Rickards H, Robertson M, Stern J (2016). Clinical effectiveness and patient perspectives of different treatment strategies for tics in children and adolescents with Tourette syndrome: a systematic review and qualitative analysis. Health Technol Assess.

[41] Groth C, Skov L, Lange T, Debes NM (2019). Predictors of the clinical course of Tourette syndrome: a longitudinal study. J Child Neurol.

